# Supplementary material for: Comparison of AAV9-driven motor neuron transduction following different CNS-directed delivery methods in mice
Source: Sci Rep. 2026 Mar 4;16:12107. doi: 10.1038/s41598-026-38039-z (PMC13076636; doi:10.1038/s41598-026-38039-z)
Supplement: Supplementary file 1 — Supplementary Material 1 [file 41598_2026_38039_MOESM1_ESM.docx]

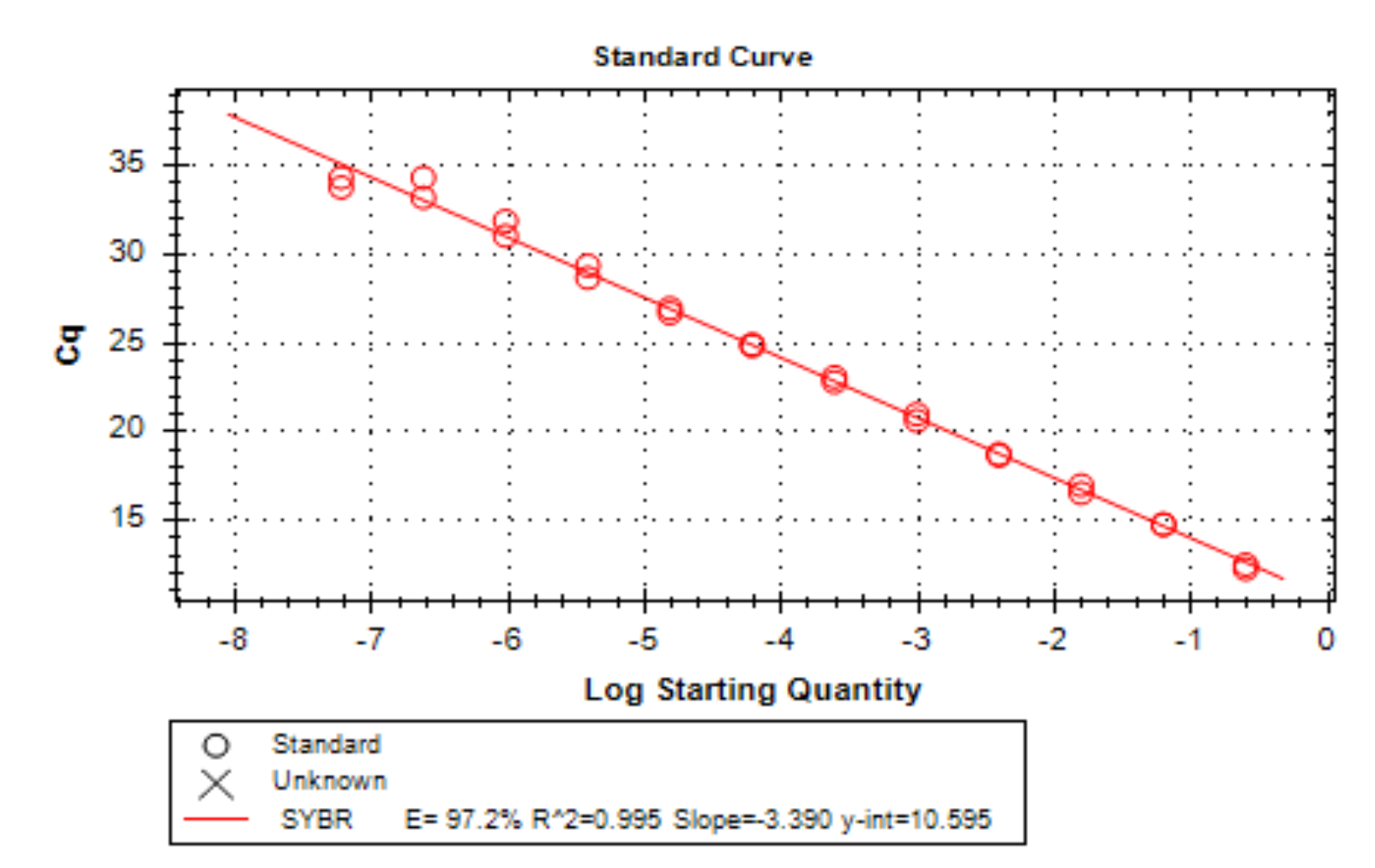


***Fig. S.1:*** ***Standard curve of CMV.GFP plasmid clone used for quantitative determination of viral genome copy number in central tissues by qPCR.***


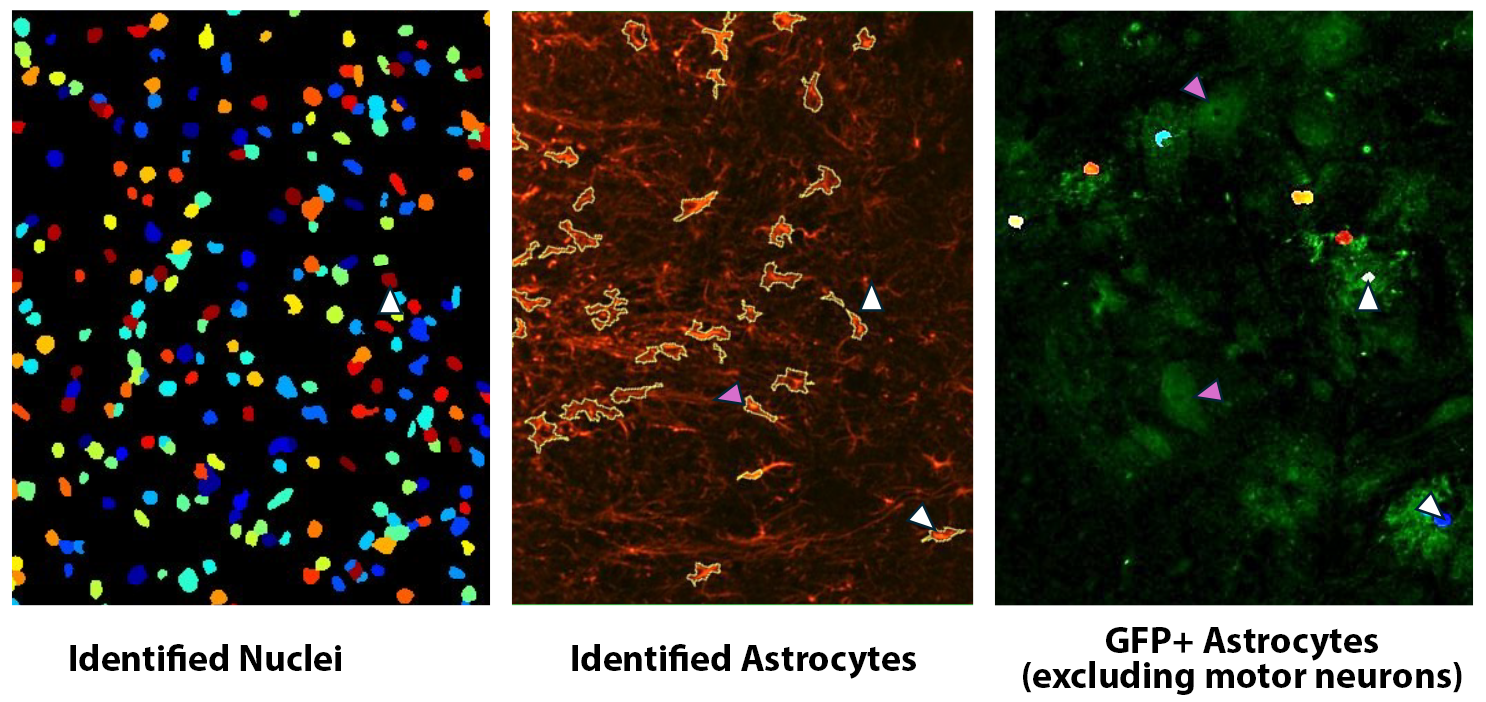


***Fig. S.2: Cell profiler mask examples showing identification of astrocytes with application of machine learning tool.*** *Note example showing the identification of an astrocyte with nucleus that co-expresses GFP (white arrow). Also note that although the motor neurons (pink arrows) express GFP, the machine was trained to exclude these from quantification.*


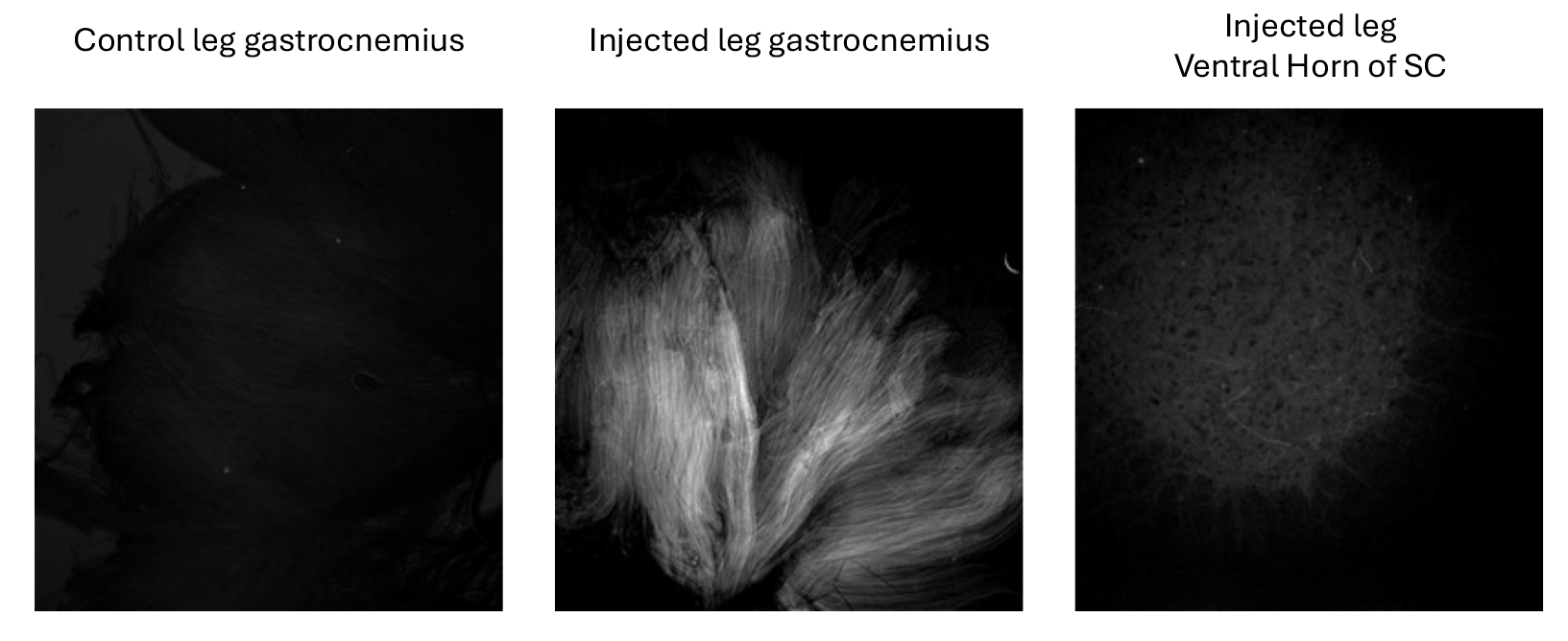


***Fig. S.3: Immunohistochemistry after intramuscular delivery of AAV9-CMV-eGFP suggested that, despite transducing myofibers, there was minimal retrograde transduction of AAV9, as GFP was not detected in lumbar spinal cord motor neurons.*** *Previous studies have similarly shown that there is minimal transduction to lumbar spinal cord after direct intramuscular hindlimb delivery of AAV9 (Jan et al., 2019, Foust et al., 2009). Intramuscular delivery of AAV9 could be useful if seeking to selectively target peripheral muscle.*

***
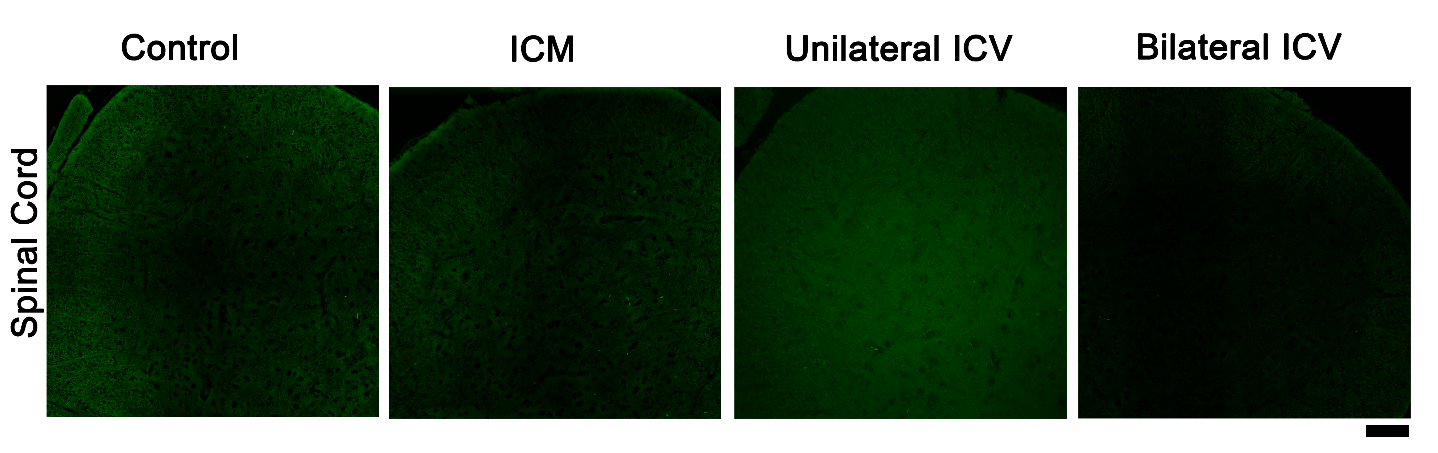
***

*­­*

***Fig S.4: No primary control immunohistochemistry for GFP staining in spinal cord in different conditions****. Note that some diffuse green fluorescence is visible at variable intensity in all groups. This could represent antibody non-specificity, or autofluorescence. True, positive staining is easily distinguishable, however this likely contributed to false positive identification during quantification. Scale bar = 50µm.*

*
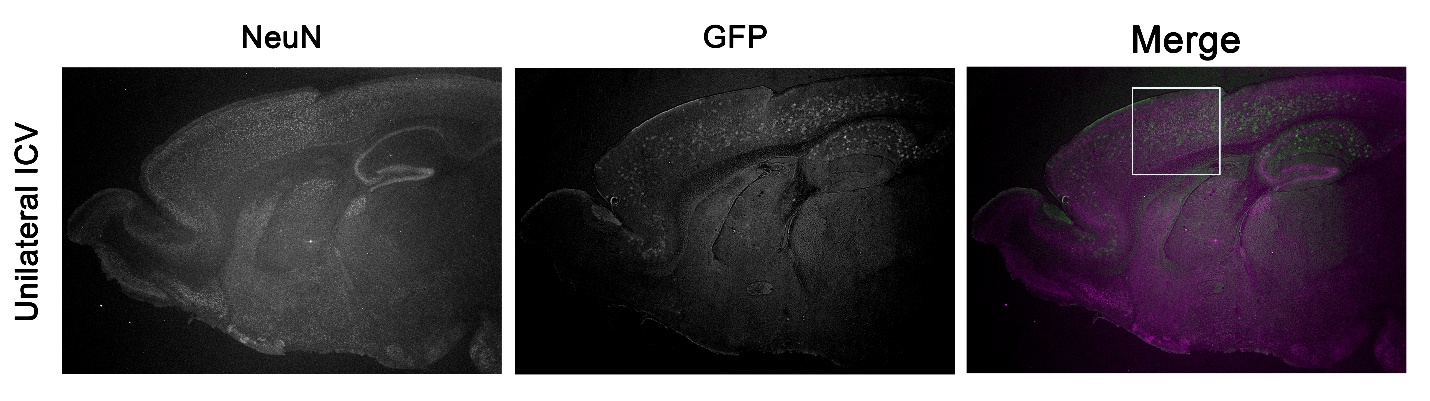
*

***Fig. S.5: Low magnification (x2) micrograph of right anterior brain hemisphere in sagittal section 4 weeks after unilateral ICV injection of AAV9-CMV-eGFP.*** *Note that some GFP positivity can be seen, however higher magnification reveals a lack of co-localisation with the neuronal marker, NeuN. The light blue box in the merged panel highlights the approximate region of interest, including motor cortex.*
